# Association Between Obesity and Histological Tumor Budding in Patients With Nonmetastatic Colon Cancer

**DOI:** 10.1001/jamanetworkopen.2021.3897

**Published:** 2021-04-01

**Authors:** Tong Gan, Kurt B. Schaberg, Daheng He, Akila Mansour, Harit Kapoor, Chi Wang, B. Mark Evers, Therese J. Bocklage

**Affiliations:** 1 Department of Surgery, The University of Kentucky, Lexington; 2 The Markey Cancer Center, The University of Kentucky, Lexington; 3 Department of Pathology, The University of Kentucky, Lexington; 4 Department of Radiology, The University of Kentucky, Lexington; 5 Department of Internal Medicine, The University of Kentucky, Lexington

## Abstract

**Importance:**

Obesity is associated with increased risk of colorectal cancer (CRC) and a more aggressive disease course. Tumor budding (TB) is an important prognostic factor for CRC, but its association with obesity is unknown.

**Objective:**

To evaluate the association of TB with obesity and other prognostic factors in colon cancer.

**Design, Setting, and Participants:**

This cohort study involved a histological review of colon cancer specimens obtained during 7 years (January 2008 to December 2015) at the University of Kentucky Medical Center; data analysis was conducted from February 2020 to January 2021. Specimens came from 200 patients with stage I to III colon cancer; patients with stage 0, stage IV, or incomplete data were excluded.

**Main Outcomes and Measures:**

TB was defined as 1 to 4 malignant cells at the invasive edge of the tumor, independently assessed by 2 academic pathologists. The primary outcome was the association of TB with obesity (defined as body mass index [BMI] of 30 or greater). Secondary outcomes include the association of TB with clinical features (ie, age, race, sex, TNM stage, tumor location) and pathological features (ie, poorly differentiated tumor clusters [PDCs], Klintrup-Mäkinen inflammatory score, desmoplasia, infiltrative tumor border, tumor necrosis, and tumor-to-stroma ratio).

**Results:**

A total of 200 specimens were reviewed. The median (interquartile range) age of patients was 62 (55-72) years, 102 (51.0%) were women, and the mean (SD) BMI was 28.5 (8.4). A total of 57 specimens (28.5%) were from stage I tumors; 74 (37.0%), stage II; and 69 (34.5%), stage III. Of these, 97 (48.5%) had low-grade (<5 buds), 36 (18.0%) had intermediate-grade (5-9 buds), and 67 (33.5%) had high-grade (≥10 buds) TB. Multivariable analysis adjusting for clinical and histological factors demonstrated that higher TB grade was associated with obesity (odds ratio [OR], 4.25; 95% CI, 1.95-9.26), higher PDC grade (grade 2 vs 1: OR, 9.14; 95% CI, 3.49-23.93; grade 3 vs 1: OR, 5.10; 95% CI, 2.30-11.27), increased infiltrative tumor border (OR, 1.03; 95% CI, 1.01-1.04), cecal location (OR, 2.55; 95% CI, 1.09-5.97), and higher stage (eg, stage III vs stage I for high-grade or intermediate-grade vs low-grade TB: OR, 2.91; 95% CI, 1.00-8.49). Additionally, patients with a higher TB grade had worse overall survival (intermediate vs low TB: hazard ratio, 2.20; 95% CI, 1.11-4.35; log-rank *P* = .02; high vs low TB: hazard ratio, 2.67; 95% CI, 1.45-4.90; log-rank *P* < .001).

**Conclusions and Relevance:**

In this cohort study, a novel association between high TB grade and obesity was found. The association could reflect a systemic condition (ie, obesity) locally influencing aggressive growth (ie, high TB) in colon cancer.

## Key Points

**Question:**

Is tumor budding associated with obesity and other histological factors associated with aggressive colon cancers?

**Findings:**

In this cohort study of 200 specimens from patients with colon cancer, high tumor budding (ie, presence of ≥5 bud consisting of 1-4 malignant cells at the invasive edge of the tumor) was significantly associated with clinical factors including obesity, higher disease stage, and cecal location. High tumor budding was also associated with poor prognostic histological features, including poorly differentiated tumor clusters and infiltrative tumor borders.

**Meaning:**

In this study, tumor budding was associated with multiple prognostic factors for aggressive tumor biology, including obesity and infiltrative tumor border.

## Teaser

This cohort study evaluates the association of tumor budding with obesity and other prognostic factors among patients with colon cancer.

## Introduction

Colorectal cancer (CRC) remains the second most common cause of cancer deaths in the United States. Approximately 52 980 people in the United States will die from CRC in 2021. The incidence among those older than 65 years has been steadily declining.^1^ However, the incidence in those aged between 50 to 64 years has been increasing by 1% annually, and that among patients younger than 50 years has been increasing by 2% annually. Similarly, mortality rates have increased by 1.3% annually in those younger than 50 years.^1,2,3^ Modifiable factors for CRC include tobacco exposure, alcohol consumption, poor diet, and obesity; these are major contributors to the increased incidence and mortality.^4^

The World Health Organization defines obesity as a body mass index (BMI; calculated as weight in kilograms divided by height in meters squared) of 30 or greater.^5,6^ During the past 40 years, the prevalence of obesity in the United States has more than doubled, accounting for 42.4% of the population in 2018.^7,8^ Obesity is a major risk factor for CRC. In men with obesity, compared with those in the normal BMI range (ie, 18.5-24.9), the relative risk of development of CRC ranges from 1.37 to 1.95.^9,10^ In women with obesity, the hazard ratio for premenopausal women is 1.88, while no association existed for postmenopausal women.^10^ Obesity is also associated with a higher risk of mortality in patients diagnosed with CRC.^11,12^ Patients with obesity have a relative risk of 1.35 to 1.84 for CRC-specific mortality compared with patients with a BMI of less than 25.^13,14^ Several proposed mechanisms of obesity-promoted cancer growth include a chronic low-grade inflammatory state, insulin resistance, altered intestinal microbiome, and more recently, unique changes in the tumor microenvironment, specifically epithelial mesenchymal transition (EMT).^15,16,17,18,19^

An emerging histologic criterion that portends a more aggressive tumor biology and worse outcomes in patients with CRC is the number of tumor buds at the invasive edge. A tumor bud is defined as 1 to 4 malignant cells at the leading edge of an epithelial tumor and has been associated with lymphovascular invasion, local recurrence, distant metastasis, and poor overall and disease-free survival, leading to its use as an independent prognostic factor for CRC.^20,21,22^ First described by Hase et al,^23^ tumor budding (TB) represents an EMT with loss of adhesion molecules, leading to increased migratory capacity and invasiveness.^24^ Currently, the quantitative method to standardize TB reporting was created by the International Tumor Budding Consensus Conference (ITBCC).^25^

Assessment of the CRC tumor microenvironment has led to several other important histological tumor features that may aid in cancer prognosis. Most prominently, these include poorly differentiated tumor clusters (PDCs) and inflammatory changes at the leading edge (ie, Klintrup-Mäkinen [KM] score). High counts of PDCs have been found to be strongly associated with nodal metastases in CRC as well as with perineural and lymphovascular invasion and poorer overall survival.^26,27,28^ Adjacent to the leading edge, peritumoral inflammation has been demonstrated to be associated with a better prognosis in CRC.^29^ Currently, the KM score is the most reproducible and prognostic inflammatory grading system in CRC.^30,31,32^ No studies have evaluated the association of TB with obesity and other histological markers of aggressive tumor growth. The objective of this study was to evaluate TB and its association with histological and clinical features, particularly obesity, in patients with colon cancer.

## Methods

### Study Design

This is a histological review of formalin-fixed colon cancer specimens collected from January 1, 2008, to December 31, 2015, at the University of Kentucky (UK) Medical Center. The sample population included patients older than 20 years who were diagnosed with cecal, ascending, transverse, descending, sigmoid, or rectosigmoid colon cancer. Patients with stage 0, stage IV, and unknown disease were excluded. Missing or inadequate tumor sections (ie, <1 section/cm of tumor) were excluded. Matched clinical and oncological outcomes data were obtained from the internal database in the UK Department of Pathology and the Kentucky Cancer Registry (KCR) oncological outcomes data. Ethical approval for use of patient specimen and KCR was obtained from the UK Office of Research Integrity institutional review board. Informed consent was obtained at the time of surgery with surgical consent for general use of specimen for future studies. It was not feasible to obtain specific consent because these samples had already been collected. The KCR is a National Cancer Institute Surveillance, Epidemiology, and End Results population-based cancer registry that has been certified by the North American Association of Central Cancer Registries for completeness, accuracy, and timeliness annually since 1997. This study followed the Strengthening the Reporting of Observational Studies in Epidemiology (STROBE) reporting guideline for observational studies.

### Definitions

Seven histological features demonstrated to be prognostic of CRC were evaluated. TB is defined as a single tumor cell or a cluster of 4 or fewer tumor cells and is graded with a 3-tier system according to the ITBCC (low, ≤4 buds; intermediate, 5-9 buds; high, ≥10 buds).^25,33^ PDCs are defined as tumor cell clusters of 5 or more cells graded with a 3-tier system (G1, ≤4 clusters; G2, 5-9 clusters; G3, ≥10 clusters).^34,35,36^ Peritumor inflammation is evaluated with the KM inflammatory Score (range, 0-3, with 0 indicating no inflammation and 3 indicating high-grade inflammation).^32^ Desmoplastic reaction, or the presence of fibrosis at the invasive front, was assessed using the Ueno scoring system.^37,38^ The amount of infiltrative tumor border was measured and reported as a percentage.^39^ The amount of tumor necrosis was measured and reported as a percentage.^40^ Tumor-to-stroma ratio was measured as the percentage of stroma within the tumor.^41^

Clinical factors evaluated included age, race, sex, tumor grade, TNM stage, tumor location, and BMI. BMI was calculated from the associated medical records at the time of tumor resection. Obesity was defined as a BMI of 30 or greater, and nonobesity was defined as a BMI of less than 30.^7^ The Kentucky cohort has among the highest number of patients below poverty level, lowest high school attainment percentage, and highest uninsured rates in the nation, resulting in significant CRC disparities. A large portion of the disparities is contributed by the eastern Appalachian portions of the state.^42^ Appalachian status was defined as patients who reside in 1 of the 54 counties in eastern Kentucky with one of the lowest education and highest poverty levels in the United States.^43^

### Tumor Sample Preparation and Scoring

All slides were reviewed by 2 subspecialty-trained, academic surgical pathologists (K.B.S. and T.J.B.). Initially, 8 colon cancer cases were reviewed simultaneously to confirm a common approach to scoring for histologic features. The pathologists followed the scoring rules reported in the literature for assessment of the 7 histological features. The pathologists used the same model microscope (Olympus BX41), objectives, and eyepieces to ensure an identical field of view diameter for scoring. The pathologists were masked to patient outcomes, treatment regimens, and BMI. All data were coded and recorded for analysis.

### Statistical Analysis

The primary analysis was to assess the association between TB and BMI, and the secondary analysis was to evaluate the association between TB and other clinical factors, histological factors, and survival time. For the primary and secondary analyses, the Fisher exact test or analysis of variance was performed to evaluate the univariate association between the TB grade and each clinical and histological factor. A multivariable partial proportional odds logistic regression for TB grade with BMI, age, sex, race, TNM stage, tumor location, Appalachian status, PDCs, desmoplasia, infiltrative tumor border, tumor-to-stroma ratio, KM inflammatory score, and tumor necrosis as explanatory variables was used to assess the association of TB with each clinical and histological factor. The adequacy of the proportional odds assumption was tested for each variable, and nonproportionality was allowed if the test was rejected. For variables that satisfied proportionality, a single common odds ratio (OR) was reported, and for variables that did not satisfy proportionality assumption, separate ORs were presented for comparing high-grade or intermediate-grade vs low-grade TB and for comparing high-grade vs intermediate-grade or low-grade grade TB. The goodness of fit of the model was assessed by using Pulkstenis-Robinson χ^2^ and deviance tests.^44^ Kaplan-Meier curves and the log-rank test were used to assess the association between the TB grade and overall survival. The Cox proportional hazards regression model was further used to adjust for covariates including age, sex, race, TNM stage, tumor location, Appalachian status, and BMI. The proportional hazards assumption was assessed using the method proposed by Grambsch and Therneau,^45^ and the goodness of fit of the model was evaluated by using the method proposed by May and Hosmer.^46^ All statistical tests were 2-sided. A *P* < .05 was considered statistically significant. Statistical analyses were performed using R version 4.0.0 (R Project for Statistical Computing), which were initiated in February 2020 and finalized in January 2021. A detailed description of data analysis procedure, R code, and R output are provided in the eAppendix in the Supplement.

## Results

### Patient Characteristics

**Figure 1.**
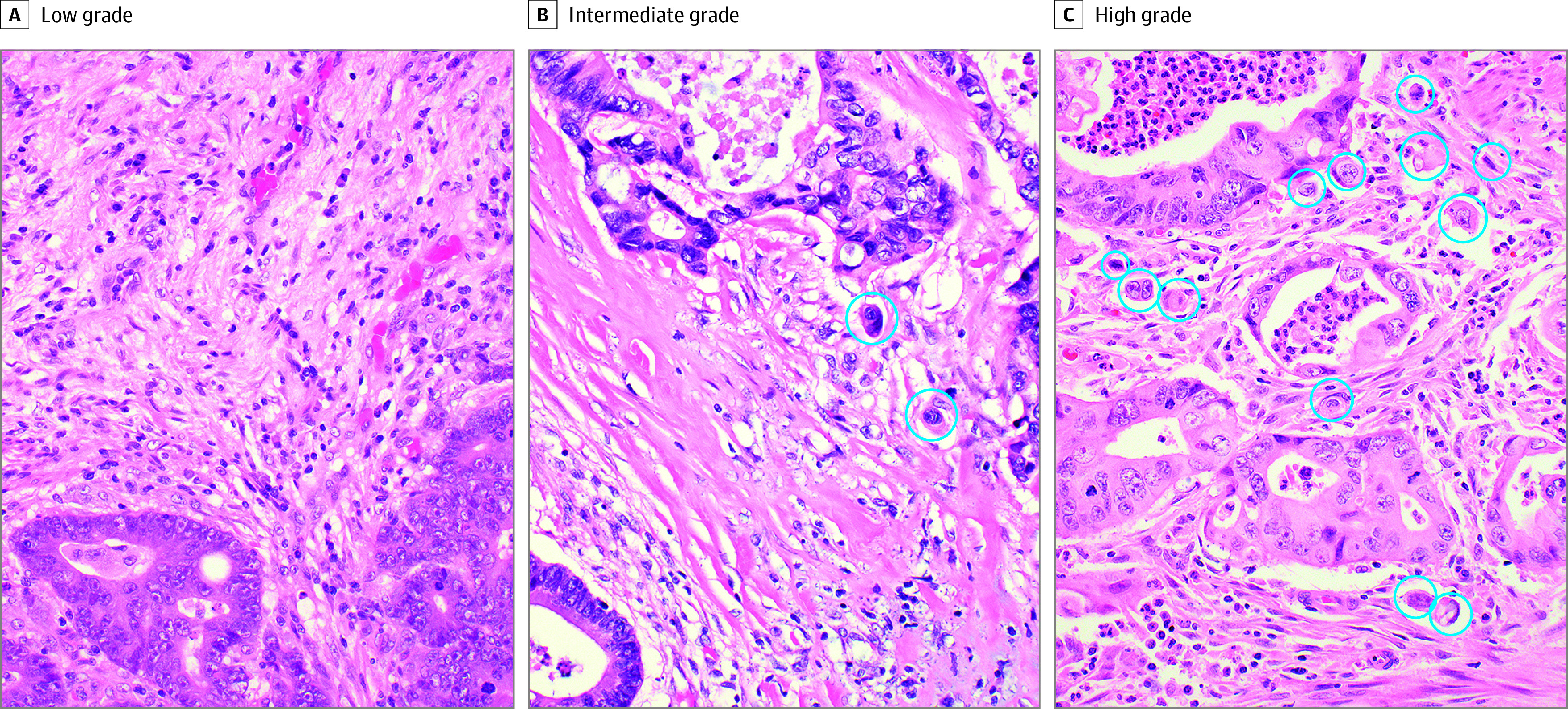
Tumor Budding Grades Samples taken from the leading edge of the tumor, cut at 4 to 5 micron thickness, stained with hematoxylin and eosin, and photographed with a ×40 objective. The actual field of view for counting is ×20 objective with a ×10 ocular field of view 22 mm, normalized by dividing by 1.21. Tumor buds are defined as tumor cells dispersed singly or in clusters of up to 4 cells. Examples of low-grade (A), intermediate-grade (B), and high-grade (C) tumor budding; tumor buds are circled.

A total of 239 colon cancer samples were identified from 2008 to 2015 at the UK. After the exclusion of 2 samples (0.8%) with stage 0 disease, 8 (3.3%) with stage IV disease, 4 (1.7%) with unknown stage, 11 (4.6%) with unknown TB grade, and 15 (6.3%) with inadequate tumor specimen for histological grading, a total of 200 samples formed our final cohort. Of these, the median (interquartile range) age of patient at diagnosis was 62 (55-72) years, and the mean (SD) BMI was 28.5 (8.4). Most patients were 75 years old or older (133 [66.5%]) (Table 1). There were nearly equal number of men (98 [49.0%]) and women (102 [51.0%]), and most were White individuals (180 [90.0%]), consistent with our state demographic characteristics.^47^ Nearly one-third of our patients had obesity (ie, BMI ≥30; 64 [32.0%]). Samples were categorized by stage: I (57 [28.5%]), II (74 [37.0%]), and III (69 [34.5%]). A total of 97 specimens (48.5%) had low-grade TB (<5 buds); 36 (18.0%), intermediate-grade TB (5-9 buds), and 67 (33.5%), high-grade TB (≥10 buds) at the invasive edge. Histological examples of each TB grade are demonstrated in Figure 1.

**Table 1.** Clinical and Histological Characteristics

Characteristic	Patients, No. (%)				*P* value^a^
	All (N = 200)	Tumor budding grade			
		Low (n = 97)	Intermediate (n = 36)	High (n = 67)	
Age, y					
<50	27 (13.5)	13 (13.4)	4 (11.1)	10 (14.9)	.78
50-74	40 (20.0)	22 (22.7)	8 (22.2)	10 (14.9)	
≥75	133 (66.5)	62 (63.9)	24 (66.7)	47 (70.1)	
Sex					
Women	102 (51.0)	50 (51.5)	15 (41.7)	37 (55.2)	.44
Men	98 (49.0)	47 (48.5)	21 (58.3)	30 (44.8)	
Race					
African American	17 (8.5)	9 (9.3)	4 (11.1)	4 (6.0)	.91
White	180 (90.0)	86 (88.7)	32 (88.9)	62 (92.5)	
Asian	2 (1.0)	1 (1.0)	0	1 (1.5)	
Unknown	1 (0.5)	1 (1.0)	0	0	
TNM stage^b^					
I	57 (28.5)	37 (38.1)	11 (30.6)	9 (13.4)	<.001
II	74 (37.0)	42 (43.3)	13 (36.1)	19 (28.4)	
III	69 (34.5)	18 (18.6)	12 (33.3)	39 (58.2)	
Tumor location					
Noncecal	163 (81.5)	85 (87.6)	28 (77.8)	50 (74.6)	.08
Cecal	37 (18.5)	12 (12.4)	8 (22.2)	17 (25.4)	
BMI					
<30	136 (68.0)	70 (72.2)	29 (80.6)	37 (55.2)	.02
≥30	64 (32.0)	27 (27.8)	7 (19.4)	30 (44.8)	
Desmoplasia					
Immature	124 (62.0)	48 (49.5)	27 (75.0)	49 (73.1)	.005
Intermediate	28 (14.0)	15 (15.5)	5 (13.9)	8 (11.9)	
Mature	48 (24.0)	34 (35.1)	4 (11.1)	10 (14.9)	
Poorly differentiated tumor clusters grade					
1	100 (50.0)	74 (76.3)	9 (25.0)	17 (25.4)	<.001
2	36 (18.0)	11 (11.3)	10 (27.8)	15 (22.4)	
3	64 (32.0)	12 (12.4)	17 (47.2)	35 (52.2)	
Infiltrative tumor border, median (range), %	60 (0-100)	35 (0-90)	65 (0-90)	85 (5-100)	<.001
Tumor to stroma ratio, median (range), %	60 (10-90)	70 (10-90)	60 (10-90)	60 (10-90)	.003
KM inflammatory score, median (range)	1 (0-3)	1 (0-3)	1 (0-3)	1 (0-3)	.39
Tumor necrosis, median (range), %	10 (0-60)	10 (0-60)	10 (0-50)	10 (0-60)	.04

Abbreviation: BMI, body mass index (calculated as weight in kilograms divided by height in meters squared).

^a^ The *P* value is for univariate analysis of the association between the clinical or histological feature and tumor budding based on the Fisher exact test for a categorical feature or analysis of variance for a continuous feature.

^b^ TNM stage based on American Joint Cancer Committee seventh edition staging system.

### Clinical Factors Associated With TB

When categorized by TB grade, higher TB grades were associated with higher BMI. For example, 30 of 67 high-grade TB samples (44.8%) had a BMI of 30 or greater compared with 27 of 97 low-grade TB samples (27.8%). In addition, higher TB grades were associated with increased tumor stage (Table 1). For example, 39 high-grade TB samples (58.2%) were stage III compared with only 18 low-grade TB samples (18.6%). Conversely, a higher number of low-grade TB samples (37 [38.1%]) were TNM stage I compared with high-grade TB samples (9 [13.4%]). TB grades were not significantly associated with age, sex, race, or tumor location in the unadjusted analysis (Table 1).

Notably, our multivariable analysis based on the partial proportional odds regression model found that patients with obesity (ie, BMI ≥30) were more likely to have higher TB grades compared with patients without obesity (ie, BMI <30) (OR, 4.25; 95% CI, 1.95-9.26; *P* < .001). Next, we identified that higher TB grades were associated with more advanced tumor stage (eg, stage III vs stage I for high-grade or intermediate-grade vs low-grade TB: OR, 2.91; 95% CI, 1.00-8.49; *P* = .04). In addition, we found that cancers arising in the cecum were more likely to be associated with higher TB grade than noncecal tumors (OR, 2.55; 95% CI, 1.09-5.97; *P* = .03) (Table 2). Within the multivariable analysis, factors not associated with TB grade included age, race, and sex (eAppendix in the Supplement).

**Table 2.** Multivariable Analysis of Clinical and Histological Factors Associated With TB

Factor	OR (95% CI)^a^	*P* value
BMI		
With or without obesity^b^	4.25 (1.95-9.26)	<.001
TNM stage^c^		
Stage II vs I		
High or intermediate vs low TB	0.83 (0.32-2.20)	.04
High vs intermediate or low TB	1.02 (0.33-3.11)	
Stage III vs I		
High or intermediate vs low TB	2.91 (1.00-8.49)	
High vs intermediate or low TB	3.32 (1.06-10.41)	
Tumor location		
Cecal vs noncecal	2.55 (1.09-5.97)	.03
Poorly differentiated tumor clusters grade		
2 vs 1	9.14 (3.49-23.93)	<.001
3 vs 1	5.10 (2.30-11.27)	
Infiltrative tumor border	1.03 (1.01-1.04)	<.001

Abbreviations: BMI body mass index (calculated as weight in kilograms divided by height in meters squared); OR, odds ratio; TB, tumor budding.

^a^ A partial proportional odds logistic model for TB was used with BMI, age, sex, race, TNM stage, tumor location, Appalachian status, poorly differentiated tumor clusters, desmoplasia, infiltrative tumor border, tumor-to-stroma ratio, Klintrup-Mäkinen inflammatory score, and tumor necrosis as explanatory variables. Proportional odds were assumed for all explanatory variables except for sex, TNM stage, Klintrup-Mäkinen inflammatory score, and tumor necrosis. A Wald test was performed to assess the association between TB and each of the explanatory variables. Only variables with *P* < .05 are reported in the table. For TNM stage that showed nonproportional odds effects, 2 ORs are reported for each contrast, 1 comparing high or intermediate with low TB and the other comparing high with intermediate or low TB. For other variables that showed a proportional odds effect, a common odds ratio is reported. Detailed model specifications and complete model fitting results are provided in the eAppendix in the Supplement.

^b^ Obesity was defined as BMI of 30 or greater.

^c^ TNM stage based on American Joint Cancer Committee seventh edition staging system.

### Histological Factors Associated With TB

Our multivariable analysis based on the partial proportional odds regression model demonstrated a significant association of PDC grade with TB grade. Both grade 2 and 3 PDC were more likely to be associated with higher TB grades compared with grade 1 PDC (grade 2 vs 1: OR, 9.14; 95% CI, 3.49-23.93; grade 3 vs 1: OR, 5.10; 95% CI, 2.30-11.27) (Table 2). An increased infiltrative tumor border was also significantly associated with increased TB grade (OR, 1.03; 95% CI, 1.01-1.04). Our multivariable analysis did not demonstrate a significant association of TB with desmoplasia, tumor-to-stroma ratio, KM inflammatory score, or tumor necrosis (eAppendix in the Supplement).

### Association of TB With Survival

**Figure 2.**
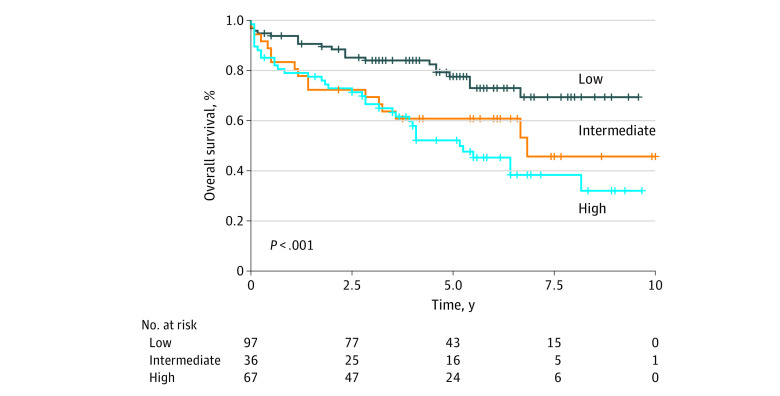
Kaplan-Meier Plot of Survival by Tumor Budding Grade Overall survival as separated by tumor budding grade (ie, low, intermediate, or high).

TB was significantly associated with survival in our study population. Analysis using Kaplan-Meier plots demonstrated that a higher TB grade was associated with worse overall survival (low-grade vs high-grade TB: hazard ratio, 2.67; 95% CI, 1.45-4.90; log-rank *P* < .001; low-grade vs intermediate-grade TB: hazard ratio, 2.20; 95% CI, 1.11-4.35; log-rank *P* = .02) (Figure 2 and eAppendix in the Supplement). Moreover, our multivariable analysis based on the Cox regression model demonstrated similar findings, with a significantly worse survival associated with an increase of TB grade (Table 3) as well as older age. Differences in tumor location, sex, race, Appalachian status, TNM stage, and BMI did not have a significant association with survival (Table 3).

**Table 3.** Multivariable Cox Proportional Hazards Regression Survival Analysis

Factor	HR (95%CI)	*P* value
Tumor budding grade		
Intermediate vs low	2.20 (1.11-4.35)	.005
High vs low	2.67 (1.45-4.90)	
Tumor location		
Cecal vs noncecal	1.02 (0.56-1.88)	.94
Age, y		
50-74 vs <50	1.30 (0.57-2.98)	<.001
≥75 vs <50	3.89 (1.59-9.50)	
Sex		
Men vs women	0.85 (0.52-1.38)	.51
Race^a^		
White vs Black	1.75 (0.60-5.10)	.30
Appalachian status		
Appalachian vs non-Appalachian	1.11 (0.67-1.84)	.69
TNM stage^b^		
Stage II vs I	1.27 (0.65-2.45)	.37
Stage III vs I	1.61 (0.82-3.14)	
BMI		
With vs without obesity^c^	1.13 (0.66-1.93)	.65

Abbreviations: BMI, body mass index (calculated as weight in kilograms divided by height in meters squared); HR, hazard ratio.

^a^ Patients with Asian or unknown race (n = 3) were excluded in this analysis.

^b^ TNM stage based on American Joint Cancer Committee seventh edition staging system.

^c^ Obesity was defined as BMI of 30 or greater.

## Discussion

The tumor microenvironment is a critical aspect of tumor biology that has provided powerful prognostic markers that complement the traditional tumor grade and staging system. Several histological features of the leading edge of the tumor are associated with worse CRC outcomes, including, most prominently, TB. We found that TB was associated with more aggressive (ie, higher stage) colon cancers and with obesity and cecal location as well as other histological prognostic factors, including PDCs and infiltrative tumor border. Importantly, we also showed that higher TB was associated with worse overall survival in our cohort, consistent with previous studies.

With the continued increasing rates of obesity in the United States, CRC incidence and mortality in those younger than 50 years have been concurrently increasing at an alarming rate.^2,3,48^ Obesity is likely a major contributor, considering that it has been demonstrated to increase the risk of early-onset CRC.^49,50^ Early-onset CRC tends to be a higher stage and more poorly differentiated at the time of initial diagnosis, but 50% of early-onset CRCs remain sporadic and unexplained by hereditary or familial predisposition.^51^ Several obesity-related mechanisms have been postulated to induce malignant cell transformation, including insulin resistance, growth factor and steroid hormone dysregulation, and chronic inflammation.^11,52^ We found that colon cancer samples with higher grades of TB were more than twice as likely to come from a patient with obesity, further reinforcing the role that obesity plays in early-onset colon cancer. One possible explanation may be that obesity has been identified as a risk factor for cells undergoing EMT through a leptin-associated pathway.^53,54^ Similarly, the histological finding of TB has been described as portions of the tumor undergoing EMT, likely related to β-catenin dysregulation.^20,55,56^ TB has also been described as having very similar properties as groups of cancer stem cells (CSCs) and may represent a subset of migrating CSCs. Increased invasiveness, chemoresistance, and tumorigenicity are all properties of CSCs that are observed in CRC.^20,57^ The adipose tissue of patients with obesity has been found to demonstrate a complex interplay within the tumor microenvironment to stimulate CSCs, thus providing another potential connection between TB and obesity.^58^ More molecular studies are needed to better elucidate the association between TB and obesity, but to our knowledge, our study is the first to identify this association.

Among other high-risk histological features, we identified an association between higher grades of TB and PDCs. The major difference between the 2 grading systems is that a tumor bud is defined as a group of fewer than 5 cells whereas a PDC is a larger cluster of at least 5 cells, thus more easily seen on hematoxylin and eosin stain.^59^ Like TB, PDCs are independent prognostic factors for CRC.^26,27,28^ Studies that use both TB and PDCs prior to standard reporting of TB are rare, but Lee et al^59^ demonstrated that TB and PDCs have high concordance in evaluating stage II colon cancers. Similarly, our results demonstrated that PDC grade 2 or 3 tumors have a significantly higher association with higher-grade TB compared with PDC grade 1 tumors. PDCs, like TB, show evidence of EMT and may even represent different phases of the same tumor growth.^60^ Our results also indicate that the 2 grading systems are very similar and may eventually result in the merging of the 2 systems.

A higher amount of infiltrative type of tumor borders also has been associated with a more aggressive tumor biology, poor CRC survival, and early recurrence.^39^ We found a significant association between TB and infiltrative tumor border. TB is often seen superimposed over the infiltrative tumor border, but because TB is observed only at high magnification, it is not used to define an infiltrative tumor border.^39,61^ Nevertheless, infiltrative tumor borders have been shown to be predictive of TB and have a synergistic effect in predicting mortality.^62^ We did not identify a statistically significant association between desmoplastic reaction and TB. Desmoplastic reaction at the tumor border and a low tumor-to-stroma ratio are other important prognostic factors for CRC survival. Cancer-associated fibroblasts that contribute to peritumoral desmoplasia also promote tumor growth through stimulating EMT and CSCs.^38^ Because TB has been postulated to comprise tumor cells undergoing EMT, there may be a complex interplay between TB and cancer-associated fibroblasts.^63^

Interestingly, we found that cecal cancers were associated with higher TB grades. Landau et al^64^ demonstrated cecal adenocarcinomas have higher levels of *KRAS* alterations and unfavorable histopathological features, including, notably, TB. Similarly, others have reported^65,66,67^ that cecal cancers tend to be more aggressive, have higher T stage, tumor grade, and TB grade, resulting in worse survival compared with left-sided CRCs. Additionally, right-sided colon cancers tend to be mismatch repair deficient, which has been shown to be associated with higher grade TB.^68^ In fact, TB has been found to provide prognostic information for mismatch repair deficient tumors with distant metastasis.^69^ Unfortunately, our database was not able to obtain accurate mismatch repair status for comparison. Lastly, we found higher TNM stage was associated with higher grades of TB.^23^ Consistent with these findings, a higher grade of TB at the invasive edge also portends worse survival.^25,70^ Interestingly, patients with stage II CRC with high-grade TB had a similar survival as those with stage III disease, indicating the significant potential association of TB with survival.^71^ As a result, TB grade has become a marker of interest for adjuvant chemotherapy in stage II colon cancer.^72^

### Limitations

Although several of our findings are consistent with previous studies regarding the prognostic value of TB in our patient population, limitations remain. Our sample population, similar to that of the commonwealth of Kentucky, is largely made of White patients, so differences based on race may not be generalizable to populations with patients from other racial/ethnic groups. The sample of our study captures all patients with stage I to III colon cancer from 2008 to 2015 in the commonwealth of Kentucky, and we were limited by the quality of the tumor specimens before 2008. All tumor samples were scored by 2 pathologists. Reproducibility and prognostic associations of TB and PDC may potentially be enhanced by immunohistochemistry or quantitative image analysis to identify tumor cells otherwise obscured by inflammation or reactive stroma. However, at this time, results have been mixed with these techniques.^73^ Furthermore, our results may not necessarily extrapolate to rectal cancers. Samples that received neoadjuvant therapy have an unknown association with TB and other histological features. Comparison with colon cancer specimens that did not receive any neoadjuvant therapy would not provide accurate results.

## Conclusions

This study found that high-grade TB was associated with obesity, cecal location, and survival. Furthermore, TB was associated with other prognostic indicators, such as PDCs and infiltrative tumor border configuration. These findings suggest that TB grade may have important implications for the prognosis of patients with colon cancer. The novel finding of the association between obesity and high TB grade may contribute to the etiology behind the trend of early-onset colon cancer. Further mechanistic evaluations are needed to identify the direct link between TB and obesity at the molecular level.

## Notes

Supplement. **eAppendix.** Supplementary Methods
