# Supplementary material for: Association Between Obesity and Histological Tumor Budding in Patients With Nonmetastatic Colon Cancer
Source: JAMA Netw Open. 2021 Apr 1;4(4):e213897. doi: 10.1001/jamanetworkopen.2021.3897 (PMC8017472; doi:10.1001/jamanetworkopen.2021.3897)
Supplement: Supplement. — eAppendix. Supplementary Methods [file jamanetwopen-e213897-s001.pdf]

Supplemental Online Content

Gan T, Schaberg KB, He D, et al. Association Between Obesity and Histological Tumor Budding in Patients With Non-metastatic Colon Cancer. *JAMA Netw Open*. 2021;4(4):e213897. doi:10.1001/jamanetworkopen.2021.3897

eAppendix. Supplementary Methods

This supplemental material has been provided by the authors to give readers additional information about their work.

© 2021 Gan T et al. *JAMA Network Open.*

eAppendix. Supplementary Methods

A. Statistical Analyses Procedure and R Output

Note: This section was written using R Markdown, which weaves together R code, the resulting R output and documentation to ensure full reproducibility of data analyses. For better readability, only the key part of the R code was presented this section The complete R code (including code for data processing, data analysis, and auxiliary functions) was provided in Section B.

1. Demographic and clinical characteristics (Table 1)

We generated descriptive statistics on patients' clinical and histological characteristics. We also compared those characteristics across different levels of tumor budding (low, intermediate or high), where a Fisher's exact test was used for comparing a categorical chracteristic and ANOVA was used for comparing a continuous characteristic.

```
vars.demo <- c("Age", "Gender", "Race", "Stage", "TumorLocation",
               "BMI", "Desmoplasia", "TumorClusters", "TumorBorder", "TumorStromaRatio",
               "KMScore", "Necrosis")
vars.type <- c("C", "C", "C", "C", "C",
               "C", "C", "C", "N", "N", "N", "N")

for (var_id in 1:length(vars.demo)) {

  if(vars.type[var_id]=="C"){
    table.self <- table(data.all[ ,vars.demo[var_id]])
    table.cross <- table(data.all[ ,c(vars.demo[var_id], "TumorBudding")])
    percent.cross <- round(t(t(table.cross) / colSums(table.cross))*100,digits=1)
    fisher.result <- fisher.test(table.cross)
    cat(paste(vars.demo[var_id], ":", sep = ""))
    print(table.self)
    print(round(table.self/sum(table.self)*100, digits=1))
    cat("\n")
    cat(paste(vars.demo[var_id], " X ", "TumorBudding Table (number):", "\n", sep = ""))
    print(table.cross)
    cat(paste(vars.demo[var_id], " X ", "TumorBudding Table (percentage):", "\n", sep = ""))
    print(percent.cross)
    cat("Fisher exact test p-value:")
    cat(round(fisher.result$p.value,digits=4), "\n\n")
  } else{
    cat(paste(vars.demo[var_id], ":\n", sep = ""))
    cat("median(min,Max): ")
    cat(paste(median(data.all[ ,vars.demo[var_id]], na.rm = T), "(",
              min(data.all[ ,vars.demo[var_id]], na.rm = T), ",",
              max(data.all[ ,vars.demo[var_id]], na.rm = T), ")", sep = ""))
    fit.anova <- aov(data.all[ ,vars.demo[var_id]] ~ data.all[ ,"TumorBudding"])
    p.anova <- round(summary(fit.anova)[[1]]$Pr[1],digits=4)
    cat("\n")
    cat("ANOVA p-value:")
```

© 2021 Gan T et al. *JAMA Network Open.*

```
    cat(p.anova, "\n\n")
    data.i.tumorbudding.1 <- data.all[data.all[ ,"TumorBudding"]==1,vars.demo[var_id]]
    data.i.tumorbudding.2 <- data.all[data.all[ ,"TumorBudding"]==2,vars.demo[var_id]]
    data.i.tumorbudding.3 <- data.all[data.all[ ,"TumorBudding"]==3,vars.demo[var_id]]
    cat(paste(vars.demo[var_id], " within TumorBudding 1:\n", sep = ""))
    cat("median(min,Max): ")
    cat(paste(median(data.i.tumorbudding.1, na.rm = T), "(",
              min(data.i.tumorbudding.1, na.rm = T), ",",
              max(data.i.tumorbudding.1, na.rm = T), ")", sep = ""))
    cat("\n\n")
    cat(paste(vars.demo[var_id], " within TumorBudding 2:\n", sep = ""))
    cat("median(min,Max): ")
    cat(paste(median(data.i.tumorbudding.2, na.rm = T), "(",
              min(data.i.tumorbudding.2, na.rm = T), ",",
              max(data.i.tumorbudding.2, na.rm = T), ")", sep = ""))
    cat("\n\n")
    cat(paste(vars.demo[var_id], " within TumorBudding 3:\n", sep = ""))
    cat("median(min,Max): ")
    cat(paste(median(data.i.tumorbudding.3, na.rm = T), "(",
              min(data.i.tumorbudding.3, na.rm = T), ",",
              max(data.i.tumorbudding.3, na.rm = T), ")", sep = ""))
    cat("\n\n")
  }


}
```

```
## Age:
##    <50   >=75 50-74
##     27     40   133
##
##    <50   >=75 50-74
##   13.5   20.0  66.5
##
##
## Age X  TumorBudding Table (number):
              TumorBudding
## Age        1   2   3
##    <50     13   4  10
##    >=75   22   8  10
##    50-74  62  24 47
## Age X TumorBudding  Table  (percentage):
##            TumorBudding
## Age           1      2      3
##    <50    13.4  11.1  14.9
##    >=75   22.7  22.2  14.9
##    50-74  63.9  66.7  70.1
## Fisher exact test p-value:0.7797
##
## Gender:
## Female     Male
##      102       98
##
## Female     Male
##       51       49
##
## Gender X TumorBudding Table (number):
```

© 2021 Gan T et al. *JAMA Network Open.*

```
##              TumorBudding
## Gender       1   2   3
##    Female 50 15 37
##    Male     47 21  30
## Gender X TumorBudding Table (percentage):
##              TumorBudding
## Gender          1      2      3
##    Female 51.5 41.7 55.2
##    Male    48.5 58.3  44.8
## Fisher exact test p-value:0.4367
##
## Race:
##    Black  Chinese  Unknown    White
##       17          2           1        180
##
##    Black  Chinese  Unknown    White
##      8.5        1.0        0.5       90.0
##
## Race X TumorBudding Table (number):
##               TumorBudding
## Race          1   2   3
##    Black       9   4   4
##    Chinese   1   0   1
##    Unknown  1   0   0
##    White     86 32  62
## Race X TumorBudding Table (percentage):
##               TumorBudding
## Race              1      2      3
##    Black      9.3  11.1   6.0
##    Chinese  1.0   0.0   1.5
##    Unknown  1.0   0.0   0.0
##    White     88.7 88.9  92.5
## Fisher exact test p-value:0.9108
##
## Stage:
##   1   2   3
## 57 74 69
##
##     1      2      3
## 28.5 37.0 34.5
##
## Stage X TumorBudding Table (number):
##         TumorBudding
## Stage   1   2   3
##       1  37  11   9
##       2 42 13 19
##       3 18 12 39
## Stage X TumorBudding Table (percentage):
##         TumorBudding
## Stage      1      2      3
##       1 38.1 30.6 13.4
##       2 43.3 36.1 28.4
##       3 18.6 33.3 58.2
## Fisher exact test p-value:0
```

© 2021 Gan T et al. *JAMA Network Open.*

```
##
## TumorLocation:
##     cecum noncecum
##        37       163
##
##     cecum noncecum
##      18.5      81.5
##
## TumorLocation X TumorBudding Table (number):
##                 TumorBudding
## TumorLocation   1  2  3
##       cecum     12  8 17
##       noncecum 85 28 50
## TumorLocation X TumorBudding Table (percentage):
##                 TumorBudding
## TumorLocation     1     2     3
##       cecum     12.4 22.2  25.4
##       noncecum 87.6 77.8 74.6
## Fisher exact test p-value:0.0812
##
## BMI:
## nonobese    Obese
##       136        64
##
## nonobese    Obese
##        68        32
##
## BMI X TumorBudding Table (number):
##             TumorBudding
## BMI            1  2  3
##   nonobese 70 29 37
##   Obese     27  7 30
## BMI X TumorBudding Table (percentage):
##             TumorBudding
## BMI              1     2     3
##   nonobese 72.2 80.6 55.2
##   Obese     27.8 19.4  44.8
## Fisher exact test p-value:0.0157
##
## Desmoplasia:
##   1   2   3
## 124  28  48
##
##  1  2  3
## 62 14 24
##
## Desmoplasia X TumorBudding Table (number):
##              TumorBudding
## Desmoplasia  1  2  3
##            1 48 27 49
##            2 15  5  8
##            3 34  4 10
## Desmoplasia X TumorBudding Table (percentage):
##              TumorBudding
```

© 2021 Gan T et al. *JAMA Network Open.*

```
## Desmoplasia       1       2       3
##               1 49.5 75.0 73.1
##               2 15.5 13.9 11.9
##               3 35.1 11.1 14.9
## Fisher exact test p-value:0.0049
##
## TumorClusters:
##   1    2    3
## 100   36   64
##
##  1  2  3
## 50 18 32
##
## TumorClusters X TumorBudding Table (number):
##                TumorBudding
## TumorClusters   1   2   3
##               1 74   9  17
##               2 11 10 15
##               3 12 17 35
## TumorClusters X TumorBudding Table (percentage):
##                TumorBudding
## TumorClusters      1      2      3
##               1 76.3 25.0 25.4
##               2 11.3 27.8 22.4
##               3 12.4 47.2 52.2
## Fisher exact test p-value:0
##
## TumorBorder:
## median(min,Max): 60(0,100)
## ANOVA p-value:0
##
## TumorBorder within TumorBudding 1:
## median(min,Max): 35(0,90)
##
## TumorBorder within TumorBudding 2:
## median(min,Max): 65(0,90)
##
## TumorBorder within TumorBudding 3:
## median(min,Max): 85(5,100)
##
## TumorStromaRatio:
## median(min,Max): 60(10,90)
## ANOVA p-value:0.0029
##
## TumorStromaRatio within TumorBudding 1:
## median(min,Max): 70(10,90)
##
## TumorStromaRatio within TumorBudding 2:
## median(min,Max): 60(10,90)
##
## TumorStromaRatio within TumorBudding 3:
## median(min,Max): 60(10,90)
##
## KMScore:
```

© 2021 Gan T et al. *JAMA Network Open.*

```
## median(min,Max): 1(0,3)
## ANOVA p-value:0.3908
##
## KMScore within TumorBudding 1:
## median(min,Max): 1(0,3)
##
## KMScore within TumorBudding 2:
## median(min,Max): 1(0,3)
##
## KMScore within TumorBudding 3:
## median(min,Max): 1(0,3)
##
## Necrosis:
## median(min,Max):  10(0,60)
## ANOVA p-value:0.0388
##
## Necrosis within TumorBudding 1:
## median(min,Max): 10(0,60)
##
## Necrosis within TumorBudding 2:
## median(min,Max): 10(0,50)
##
## Necrosis within TumorBudding 3:
## median(min,Max): 10(0,60)
```

2. Associations between tumor budding and clinical/histological features (Table 2)

As tumor budding is ordinal (low, intermediate, high), we utilized the class of cumulative logit models. A popular model in the class is the proportional odds model. To examine the applicability of the proportional odds model to our data, we first utilized a fully nonproportional odds model for tumor budding with BMI, age, gender, race, TNM stage, tumor location, Appalachian status, poorly differentiated tumor clusters, desmoplasia, infiltrative tumor border, tumor to stroma ratio, KM inflammatory score and tumor necrosis as explanatory variables. Based on this model, we assessed the proportional odds assumption for each of the explanatory variables by testing whether the model parameters for an explanatory variable across the logits are the same based on a Wald test. Note that Patients with Chinese or unknown race (n=3) were excluded in this analysis.

```r
library(ordinal)
nonPO.fit <- clm(TumorBudding ~ 1,
                 nominal = ~  BMI + Age + Gender + Race + Stage + TumorLocation  +
              AppalachianStatus + TumorClusters + Desmoplasia + TumorBorder +
              TumorStromaRatio + KMScore + Necrosis, data=data.use)
variables = c("BMI", "Age", "Gender", "Race", "Stage", "TumorLocation",
         "AppalachianStatus", "TumorClusters", "Desmoplasia", "TumorBorder",
         "TumorStromaRatio", "KMScore", "Necrosis")
PO.pval = matrix(nrow=length(variables),ncol=1)
rownames(PO.pval) = variables; colnames(PO.pval) = "p-value"
for (i in 1:length(variables)){
  PO.pval[i] = check_PO(vcov = vcov(nonPO.fit),
                        coef.beta = coef(nonPO.fit),
                        coef.name =  variables[i])
}
print(PO.pval)
```

© 2021 Gan T et al. *JAMA Network Open.*

```
## BMI                 0.1333
## Age                 0.7257
## Gender              0.0490
## Race                0.6327
## Stage               0.0180
## TumorLocation       0.8323
## AppalachianStatus   0.0821
## TumorClusters       0.0921
## Desmoplasia         0.1779
## TumorBorder         0.6020
## TumorStromaRatio    0.6620
## KMScore             0.0293
## Necrosis            0.0492 ##   p-value
```

Based on the above test, the proportional odds assumption appeared to hold for most explanatory variables (non-significant p-values) except for gender, TNM stage, KM inflammatory score and tumor necrosis (significant p-values). Therefore, we fitted the following partial proportional odds logistic model, which assumed proportional odds effects for BMI, age, race, tumor location, Appalachian status, poorly differentiated tumor clusters, desmoplasia, infiltrative tumor border, and tumor to stroma ratio, and non-proportional odds effects for gender, TNM stage, KM inflammatory score and tumor necrosis.

```
partialPO.fit <- clm(TumorBudding ~ BMI + Age + Race + TumorLocation +
                     AppalachianStatus + TumorClusters + Desmoplasia + TumorBorder +
                     TumorStromaRatio,
                     nominal = ~ Gender + Stage + KMScore + Necrosis, data=data.use)
print(summary(partialPO.fit))
```

```
## formula:
## TumorBudding ~ BMI + Age + Race + TumorLocation + AppalachianStatus +
TumorClusters + Desmoplasia + TumorBorder + TumorStromaRatio
## nominal: ~Gender + Stage + KMScore + Necrosis
## data: data.use
##
## link threshold nobs logLik AIC niter max.grad cond.H
## logit flexible 194 -144.09 336.19 6(0) 2.89e-11 9.6e+05
##
## Coefficients:
## Estimate Std. Error z value Pr(>|z|)
## BMIObese 1.447263 0.397438 3.641 0.000271 ***
## Age>=75 -0.117708 0.662995 -0.178 0.859085
## Age50-74 0.388058 0.566052 0.686 0.492996
## RaceWhite -0.124392 0.660396 -0.188 0.850594
## TumorLocationcecum 0.934632 0.435025 2.148 0.031678 *
## AppalachianStatusApp 0.433238 0.344425 1.258 0.208443
## TumorClusters2 2.212302 0.491289 4.503 6.70e-06 ***
## TumorClusters3 1.628388 0.404964 4.021 5.79e-05 ***
## Desmoplasia2 -1.065458 0.483172 -2.205 0.027445 *
## Desmoplasia3 -0.660757 0.474347 -1.393 0.163625
## TumorBorder 0.026863 0.006605 4.067 4.76e-05 ***
## TumorStromaRatio 0.003584 0.008671 0.413 0.679318
## ---
## Signif. codes: 0 '***' 0.001 '**' 0.01 '*' 0.05 '.' 0.1 ' ' 1
##
## Threshold coefficients:
```

© 2021 Gan T et al. *JAMA Network Open.*

```
## Estimate Std. Error z value
##  1|2.(Intercept)  3.761588  1.250816  3.007
##  2|3.(Intercept)  4.682715  1.293688  3.620
## 1|2.GenderMale -0.534194 0.390993 -1.366
## 2|3.GenderMale 0.096174 0.397799 0.242
## 1|2.Stage2 0.181677 0.495803 0.366
##  2|3.Stage2  -0.020261  0.568748 -0.036
##  1|2.Stage3  -1.068812  0.545701 -1.959
##  2|3.Stage3  -1.199386  0.583528 -2.055
## 1|2.KMScore -0.086169 0.272475 -0.316
## 2|3.KMScore 0.247880 0.339649 0.730
## 1|2.Necrosis 0.009187 0.017091 0.538
## 2|3.Necrosis -0.007203 0.017016 -0.423
## (3 observations deleted due to missingness)
```

We performed the Pulkstenis-Robinson goodness-of-fit tests, which supported the adequacy of the model.

```
library(generalhoslem)
```

```
## Loading required package:  reshape
## Loading required package:  MASS
```

```
pulkrob.chisq(partialPO.fit,  variables)
```

```
##
## Pulkstenis-Robinson chi-squared test
##
## data: formula: TumorBudding ~ BMI + Age + Race +
TumorLocation + AppalachianStatus + formula: TumorClusters
+ Desmoplasia + TumorBorder + TumorStromaRatio
## X-squared = 365.91, df = 370, p-value = 0.5503
```

```
pulkrob.deviance(partialPO.fit,   variables)
```

```
##
## Pulkstenis-Robinson deviance test
##
## data: formula: TumorBudding ~ BMI + Age + Race +
TumorLocation + AppalachianStatus + formula: TumorClusters
+ Desmoplasia + TumorBorder + TumorStromaRatio
## Deviance-squared = 288.19, df = 370, p-value = 0.9994
```

Based on this partial proportional odds logistic model, we performed a Wald test to assess the association between tumor budding and each of the clinical/histological features.

```
pvalue = NULL
for (i in 1:length(variables)){

  if (variables[i] %in% c("Gender", "Stage", "KMScore", "Necrosis")){
    pv.ci.fit <- Wald_pvalue_CI(vcov = vcov(partialPO.fit),
                                coef.beta = coef(partialPO.fit),
                                coef.name = variables[i], nominal = T)
  } else{
    pv.ci.fit <- Wald_pvalue_CI(vcov =  vcov(partialPO.fit),
                                coef.beta = coef(partialPO.fit),
                                coef.name =  variables[i])
  }
```

© 2021 Gan T et al. *JAMA Network Open.*

```r
print(variables[i])
print(pv.ci.fit)
pvalue[i] = pv.ci.fit$p_value
}
```

```
## [1] "BMI"
## $p_value
## [1] 3e-04
##
## $estimation
##      point_estimate CI_lower CI_upper
## [1,]           4.25     1.95     9.26
##
## [1] "Age"
## $p_value
## [1] 0.4444
##
## $estimation
##      point_estimate CI_lower CI_upper
## [1,]           0.89     0.24     3.26
## [2,]           1.47     0.49     4.47
##
## [1] "Gender"
## $p_value
## [1] 0.1969
##
## $estimation
##      point_estimate CI_lower CI_upper
## [1,]           1.71     0.79     3.67
## [2,]           0.91     0.42     1.98
##
## [1] "Race"
## $p_value
## [1] 0.8506
##
## $estimation
##      point_estimate CI_lower CI_upper
## [1,]           0.88     0.24     3.22
##
## [1] "Stage"
## $p_value
## [1] 0.0376
##
## $estimation
##      point_estimate CI_lower CI_upper
## [1,]           0.83     0.32     2.20
## [2,]           1.02     0.33     3.11
## [3,]           2.91     1.00     8.49
## [4,]           3.32     1.06    10.41
##
## [1] "TumorLocation"
## $p_value
## [1] 0.0317
##
```

© 2021 Gan T et al. *JAMA Network Open.*

```
## $estimation
##      point_estimate CI_lower CI_upper
## [1,]           2.55     1.09     5.97
##
## [1] "AppalachianStatus"
## $p_value
## [1] 0.2084
##
## $estimation
##      point_estimate CI_lower CI_upper
## [1,]           1.54     0.79     3.03
##
## [1] "TumorClusters"
## $p_value
## [1] 0
##
## $estimation
##      point_estimate CI_lower CI_upper
## [1,]           9.14     3.49    23.93
## [2,]           5.10     2.30    11.27
##
## [1] "Desmoplasia"
## $p_value
## [1] 0.051
##
## $estimation
##      point_estimate CI_lower CI_upper
## [1,]           0.34     0.13     0.89
## [2,]           0.52     0.20     1.31
##
## [1] "TumorBorder"
## $p_value
## [1] 0
##
## $estimation
##      point_estimate CI_lower CI_upper
## [1,]           1.03     1.01     1.04
##
## [1] "TumorStromaRatio"
## $p_value
## [1] 0.6793
##
## $estimation
##      point_estimate CI_lower CI_upper
## [1,]              1     0.99     1.02
##
## [1] "KMScore"
## $p_value
## [1] 0.5809
##
## $estimation
##      point_estimate CI_lower CI_upper
## [1,]           1.09     0.64     1.86
## [2,]           0.78     0.40     1.52
```

```
##
## [1] "Necrosis"
## $p_value
## [1] 0.6264
##
## $estimation
##      point_estimate CI_lower CI_upper
## [1,]           0.99     0.96     1.02
## [2,]           1.01     0.97     1.04
```

```
names(pvalue) = variables
print(pvalue)
```

```
##              BMI              Age           Gender             Race
##           0.0003           0.4444           0.1969           0.8506
##            Stage    TumorLocation AppalachianStatus   TumorClusters
##           0.0376           0.0317           0.2084           0.0000
##      Desmoplasia      TumorBorder TumorStromaRatio          KMScore
##           0.0510           0.0000           0.6793           0.5809
##         Necrosis
##           0.6264
```

Based on the above analysis, we identified significant associations between tumor budding and BMI, stage, tumor location, poorly differentiated tumor clusters and infiltrative tumor border. Note that for BMI, tumor location, poorly differentiated tumor clusters and infiltrative tumor border, a single odds ratio was reported because the proportional odds assumption held. For TNM stage, two separate odds ratios were reported for each level of stage because the proportional odds assumption was not satisfied for this variable.

3. Association between tumor budding and survival time (Figure 1 and Table 3)

We first used Kaplan-Meier curves and logrank test to assess the association between tumor budding and survival time.

```
library(survival)
library(survminer)
```

```
## Loading required package: ggplot2
## Loading required package: ggpubr
```

```
fit <- survfit(Surv(Survtime, Status) ~ TumorBudding, data=data.use1)
temp <- survdiff(Surv(Survtime, Status) ~ TumorBudding, data=data.use1)
thispvalue <- (1 - pchisq(temp$chisq, length(temp$n) - 1))
cat("Logrank P for comparing survival time across tumor budding groups:",
    round(thispvalue,digits=5), "\n\n")
```

```
## Logrank P for comparing survival time across tumor budding groups: 0.00058
```

```
ggsurvplot(fit, data=data.use1, risk.table = TRUE, pval=T,
           legend.labs=c("Low", "Intermediate", "High"),
           title="Kaplan-Meier Plot by Tumor Budding Grade",
           xlab="Time(years)", ylab="Alive (*100%)")
```

© 2021 Gan T et al. *JAMA Network Open.*

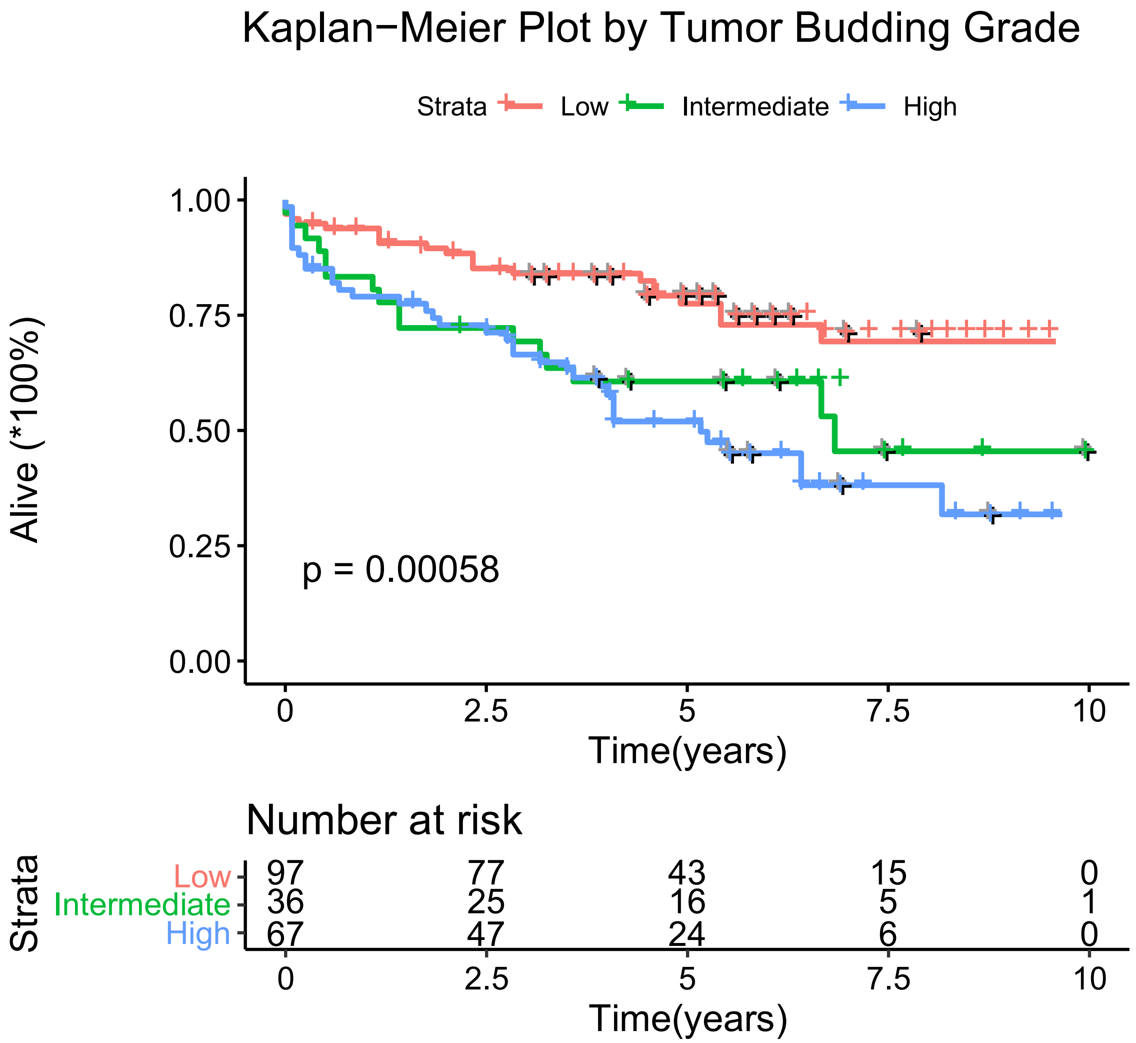

Pairwise comparisons of survival time between two levels of tumor budding were also performed using logrank tests.

```
data12 <- data.use1[data.use1$TumorBudding==1 | data.use1$TumorBudding==2, ]
fit <- survfit(Surv(Survtime, Status) ~ TumorBudding, data=data12)
temp <- survdiff(Surv(Survtime, Status) ~ TumorBudding, data=data12)
thispvalue <- (1 - pchisq(temp$chisq, length(temp$n) - 1))
cat("Logrank P for comparing survival time between intermediate vs low tumor budding:",
    round(thispvalue,digits=4), "\n\n")
```

```
## Logrank P for comparing survival time between
intermediate vs low tumor budding: 0.0232
```

```
data13 <- data.use1[data.use1$TumorBudding==1 | data.use1$TumorBudding==3, ]
fit <- survfit(Surv(Survtime, Status) ~ TumorBudding, data=data13)
temp <- survdiff(Surv(Survtime, Status) ~ TumorBudding, data=data13)
thispvalue <- (1 - pchisq(temp$chisq, length(temp$n) - 1))
cat("Logrank P for comparing survival time between high vs low tumor budding:",
    round(thispvalue,digits=4), "\n\n")
```

```
## Logrank P for comparing survival time between high vs
low tumor budding: 1e-04
```

© 2021 Gan T et al. *JAMA Network Open.*

```
data23 <- data.use1[data.use1$TumorBudding==2 | data.use1$TumorBudding==3, ]
fit <- survfit(Surv(Survtime, Status) ~ TumorBudding, data=data23)
temp <- survdiff(Surv(Survtime, Status) ~ TumorBudding, data=data23)
thispvalue <- (1 - pchisq(temp$chisq, length(temp$n) - 1))
cat("Logrank P for comparing survival time between high vs intermediate tumor budding:",
    round(thispvalue,digits=4), "\n\n")
```

```
## Logrank P for comparing survival time between high vs
intermediate tumor budding: 0.3513
```

We next used a proportional hazards model to study the association between tumor budding and survival time with adjustment for BMI, age, gender, race, TNM stage, tumor location, and Appalachian status. Note that Patients with Chinese or unknown race (n=3) were excluded in this analysis.

```
fit.cox <- coxph(Surv(Survtime, Status) ~ TumorBudding + BMI + Age + Gender + Race + Stage +
                         TumorLocation   + AppalachianStatus, data=data.use)
```

We checked the proportional hazards assumption based on the method proposed by Grambsch and Therneau (1994). As shown below, the assumption appeared to hold for all explanatory variables (non-significant p-values).

```
cox.zph(fit.cox)
```

```
##                       chisq  df    p
## TumorBudding        0.64905   2  0.72
## BMI                 0.52991   1  0.47
## Age                 4.12511   2  0.13
## Gender              0.00616   1  0.94
## Race                2.15933   1  0.14
## Stage               0.55600   2  0.76
## TumorLocation       0.10567   1  0.75
## AppalachianStatus   0.48316   1  0.49
## GLOBAL              8.42014  11  0.68
```

We also performed the goodness-of-fit test proposed by May and Hosmer (2004), which supported the adequacy of the model.

```
library(survMisc)
```

```
##
## Attaching package: 'survMisc'
## The following object is masked from 'package:ggplot2':
##
##      autoplot
```

```
print(gof(fit.cox), maxCol=20)
```

```
## $groups
## n e exp z p
## 1: <multi-column> 20 19.37962 0.14092477 0.8879294
## 2: <multi-column> 53 53.62038 -0.08472176 0.9324826
##
## $lrTest
## Analysis of Deviance Table
## Cox model: response is Surv(Survtime, Status)
## Model 1: ~ TumorBudding + BMI + Age + Gender + Race +
Stage + TumorLocation +  AppalachianStatus
```

© 2021 Gan T et al. *JAMA Network Open.*

```
## Model 2: ~ TumorBudding + BMI + Age + Gender + Race +
Stage + TumorLocation + AppalachianStatus + indicG
## loglik Chisq Df P(>|Chi|)
## 1 -337.11
## 2 -337.08 0.0694 1 0.7922
```

Based on this model, we identified a significant association between tumor budding and survival time.

```
variables = c("TumorBudding", "BMI", "Age", "Gender", "Race", "Stage", "TumorLocation",
              "AppalachianStatus")
for (i in 1:length(variables)){
  cox.pvalue.ci <- Wald_pvalue_CI(vcov = vcov(fit.cox),
                                  coef.beta = summary(fit.cox)$coefficients[ ,1],
                                  coef.name = variables[i])
  print(paste("For ", variables[i]))
  print(cox.pvalue.ci)
}
```

```
## [1] "For  TumorBudding"
## $p_value
## [1] 0.0045
##
## $estimation
##      point_estimate CI_lower CI_upper
## [1,]           2.20     1.11     4.35
## [2,]           2.67     1.45     4.90
##
## [1] "For  BMI"
## $p_value
## [1] 0.6451
## $estimation
##      point_estimate CI_lower CI_upper
## [1,]           1.13     0.66     1.93
##
## [1] "For  Age"
## $p_value
## [1] 1e-04
##
## $estimation
##      point_estimate CI_lower CI_upper
## [1,]           3.89     1.59     9.50
## [2,]           1.30     0.57     2.98
##
## [1] "For  Gender"
## $p_value
## [1] 0.5061
##
## $estimation
##      point_estimate CI_lower CI_upper
## [1,]           0.85     0.52     1.38
##
## [1] "For  Race"
## $p_value
## [1] 0.3039
```

© 2021 Gan T et al. *JAMA Network Open.*

```
##
## $estimation
##       point_estimate CI_lower CI_upper
## [1,]           1.75      0.6      5.1
##
## [1] "For   Stage"
## $p_value
## [1] 0.3668
##
## $estimation
##       point_estimate CI_lower CI_upper
## [1,]           1.27     0.65     2.45
## [2,]           1.61     0.82     3.14
## [1] "For   TumorLocation"
## $p_value
## [1] 0.9438
##
## $estimation
##       point_estimate CI_lower CI_upper
## [1,]           1.02     0.56     1.88
##
## [1] "For   AppalachianStatus"
## $p_value
## [1] 0.6932
##
## $estimation
##       point_estimate CI_lower CI_upper
## [1,]           1.11     0.67     1.84
```

References

P. Grambsch and T. Therneau (1994), Proportional hazards tests and diagnostics based on weighted residuals. Biometrika, 81, 515-26.

May S, Hosmer DW 2004. A cautionary note on the use of the Gronnesby and Borgan goodness-of-fit test for the Cox proportional hazards model. Lifetime Data Analysis 10(3):283–91.

B. Complete R Code

```
# Data processing
data.all <- read.delim(file = "2-27-20 Tumor  Bud  BMI  Final  Data  Sheet  Deidentified_use.txt",
                       header = T, stringsAsFactors = F)
data.all$TumorBorder <- as.numeric(data.all$Invasive.Front.....)
data.all$Desmoplasia <- data.all$Desmoplasia..Ueno.Score.
data.all$Desmoplasia[data.all$Desmoplasia..Ueno.Score.%in%c("4", "3,4", "4,2")] <- 3
data.all$Desmoplasia[data.all$Desmoplasia==""] = "Unknown"

data.all <- data.all[!data.all$beststagegroup%in%c("Stage 0", "Stage IV", "Stage Unknown"), ]

data.all <- data.all[(data.all$Tumor.Buds.Score..Ueno.Scheme.!=0) &
                     !is.na(data.all$Tumor.Buds.Score..Ueno.Scheme.), ]

data.all$Age <- NA
```

```r
data.all$Age[data.all$diagage<50] <- "<50"
data.all$Age[data.all$diagage>=50 & data.all$diagage<=74] <- "50-74"
data.all$Age[data.all$diagage>=75] <- ">=75"

data.all$Gender <- data.all$gender

data.all$Race <- data.all$race

data.all$AppalachianStatus <- data.all$appalachia_status


data.all$Stage <- data.all$beststagegroup
data.all$Stage[data.all$beststagegroup=="Stage I"] <- "1"
data.all$Stage[data.all$beststagegroup%in%c("Stage IIB", "Stage IIA")] <- "2"
data.all$Stage[data.all$beststagegroup%in%c("Stage IIIA", "Stage IIIB", "Stage IIIC")] <- "3"


data.all$Stage[data.all$beststagegroup.regrp=="Stage Unknown"] <- NA


data.all$Location.QC[data.all$Location.QC=="10"] <- "1"
data.all$Location.QC[data.all$Location.QC=="2,3"] <- "2"
data.all$TumorLocation <- NA
data.all$TumorLocation[data.all$Location.QC=="1"] <- "cecum"
data.all$TumorLocation[data.all$Location.QC!="1" & data.all$Location.QC!=""] <- "noncecum"
data.all$TumorLocation[data.all$Location.QC==""] <- "Unknown"


data.all$BMI.regrp <- NA
data.all$BMI.regrp[data.all$BMI < 30] <- "nonobese"
data.all$BMI.regrp[data.all$BMI >= 30] <- "Obese"
data.all$BMI <- data.all$BMI.regrp

data.all$TumorClusters <- data.all$PD.tumor.clusters

data.all$Survtime <- data.all$survmonths/12
data.all$Status <- NA
data.all$Status[grep("Alive", data.all$vital_status)] <- 0
data.all$Status[grep("Dead", data.all$vital_status)] <- 1


data.all$TumorBudding <- data.all$Tumor.Buds.Score..Ueno.Scheme.

data.all$TumorStromaRatio = data.all$Tumor.Stroma.Ratio....

data.all$KMScore = data.all$K.M.Score

data.all$Necrosis = data.all$Necrosis....

# demographic and clinical characteristics (Table 1)
vars.demo <- c("Age", "Gender", "Race", "Stage", "TumorLocation",
               "BMI", "Desmoplasia", "TumorClusters", "TumorBorder", "TumorStromaRatio",
               "KMScore", "Necrosis")
vars.type <- c("C", "C", "C", "C", "C",
               "C", "C", "C", "N", "N", "N", "N")
```

© 2021 Gan T et al. *JAMA Network Open.*

```r
for (var_id in 1:length(vars.demo)) {

  if(vars.type[var_id]=="C"){
    table.self <- table(data.all[ ,vars.demo[var_id]])
    table.cross  <- table(data.all[ ,c(vars.demo[var_id], "TumorBudding")])
    percent.cross  <- round(t(t(table.cross) / colSums(table.cross))*100,digits=1)
    fisher.result <- fisher.test(table.cross)
    cat(paste(vars.demo[var_id], ":", sep = ""))
    print(table.self)
    print(round(table.self/sum(table.self)*100,  digits=1))
    cat("\n")
    cat(paste(vars.demo[var_id], " X ", "TumorBudding Table (number):", "\n", sep = ""))
    print(table.cross)
    cat(paste(vars.demo[var_id], " X ", "TumorBudding Table (percentage):", "\n", sep = ""))
    print(percent.cross)
    cat("Fisher exact test p-value:")
    cat(round(fisher.result$p.value,digits=4), "\n\n")
  } else{
    cat(paste(vars.demo[var_id], ":\n", sep = ""))
    cat("median(min,Max): ")
    cat(paste(median(data.all[ ,vars.demo[var_id]], na.rm = T), "(",
              min(data.all[ ,vars.demo[var_id]], na.rm = T), ",",
              max(data.all[ ,vars.demo[var_id]], na.rm = T), ")", sep = ""))
    fit.anova <- aov(data.all[ ,vars.demo[var_id]] ~ data.all[ ,"TumorBudding"])
    p.anova  <-  round(summary(fit.anova)[[1]]$Pr[1],digits=4)
    cat("\n")
    cat("ANOVA p-value:")
    cat(p.anova, "\n\n")
    data.i.tumorbudding.1  <-  data.all[data.all[  ,"TumorBudding"]==1,vars.demo[var_id]]
    data.i.tumorbudding.2  <-  data.all[data.all[  ,"TumorBudding"]==2,vars.demo[var_id]]
    data.i.tumorbudding.3  <-  data.all[data.all[  ,"TumorBudding"]==3,vars.demo[var_id]]
    cat(paste(vars.demo[var_id], " within TumorBudding 1:\n", sep = ""))
    cat("median(min,Max): ")
    cat(paste(median(data.i.tumorbudding.1,  na.rm  =  T),  "(",
              min(data.i.tumorbudding.1,  na.rm  =  T),  ",",
              max(data.i.tumorbudding.1,  na.rm  =  T),  ")",  sep  =  ""))
    cat("\n\n")
    cat(paste(vars.demo[var_id], " within TumorBudding 2:\n", sep = ""))
    cat("median(min,Max): ")
    cat(paste(median(data.i.tumorbudding.2,  na.rm  =  T),  "(",
              min(data.i.tumorbudding.2,  na.rm  =  T),  ",",
              max(data.i.tumorbudding.2,  na.rm  =  T),  ")",  sep  =  ""))
    cat("\n\n")
    cat(paste(vars.demo[var_id], " within TumorBudding 3:\n", sep = ""))
    cat("median(min,Max): ")
    cat(paste(median(data.i.tumorbudding.3,  na.rm  =  T),  "(",
              min(data.i.tumorbudding.3,  na.rm  =  T),  ",",
              max(data.i.tumorbudding.3,  na.rm  =  T),  ")",  sep  =  ""))
    cat("\n\n")
  }
}

data.TumorBuddingFiltered <- data.all
```

© 2021 Gan T et al. *JAMA Network Open.*

```
data.TumorBuddingFiltered$TumorBudding <- factor(data.TumorBuddingFiltered$TumorBudding)
data.TumorBuddingFiltered$BMI <- factor(data.TumorBuddingFiltered$BMI,
                                         levels = c("nonobese", "Obese"))
data.TumorBuddingFiltered[ ,"TumorClusters"] <- factor(data.TumorBuddingFiltered[ ,"TumorClusters"],
                                                        levels = c(1,2,3))
data.TumorBuddingFiltered[ ,"Gender"] <- factor(data.TumorBuddingFiltered[ ,"Gender"],
                                                 levels = c("Female", "Male"))
data.TumorBuddingFiltered[ ,"TumorLocation"] <- factor(data.TumorBuddingFiltered[ ,"TumorLocation"],
                                                        levels = c("cecum", "noncecum"))
data.TumorBuddingFiltered$TumorLocation = relevel(data.TumorBuddingFiltered$TumorLocation,
                                                  ref="noncecum")
data.TumorBuddingFiltered[ ,"Stage"] <- factor(data.TumorBuddingFiltered[ ,"Stage"],
                                                levels = c("1", "2", "3"))
data.TumorBuddingFiltered[ ,"Desmoplasia"] <- factor(data.TumorBuddingFiltered[ ,"Desmoplasia"],
                                                      levels = c(1,2,3))
data.TumorBuddingFiltered$AppalachianStatus[data.TumorBuddingFiltered$AppalachianStatus==
                                             "Appalachian county"] <- "App"
data.TumorBuddingFiltered$AppalachianStatus[data.TumorBuddingFiltered$AppalachianStatus
                                            =="not Appalachian county"] <- "nonApp"
data.TumorBuddingFiltered$AppalachianStatus <- factor(data.TumorBuddingFiltered$AppalachianStatus,
                                                       levels = c("nonApp", "App"))
data.use1 = data.TumorBuddingFiltered
data.TumorBuddingFiltered = data.TumorBuddingFiltered[(data.TumorBuddingFiltered$Race != "Unknown") &
                                                       (data.TumorBuddingFiltered$Race!="Chinese"), ]
data.TumorBuddingFiltered[ ,"Race"] <- factor(data.TumorBuddingFiltered[ ,"Race"])

clinical.factors <- c("BMI", "Age", "Gender", "Race", "Stage", "TumorLocation","AppalachianStatus")

histological.factors <- c("TumorClusters", "Desmoplasia", "TumorBorder",
                          "TumorStromaRatio", "KMScore", "Necrosis")


data.use <- data.TumorBuddingFiltered[,c("TumorBudding",clinical.factors,
                                         histological.factors, "Survtime", "Status")]

# Associations between  tumor budding  and clinical/histological features (Table 2)
# Auxiliary functions
Wald_pvalue_CI <- function(vcov, coef.beta, coef.name, nominal=FALSE)
{
  vcov.use = vcov[grep(coef.name, row.names(vcov)),grep(coef.name, colnames(vcov))]
  if(class(coef.beta)[1]=="numeric") {coef.beta.use =
    coef.beta[grep(coef.name, names(coef.beta))];}
  if(class(coef.beta)[1]=="matrix") {coef.beta.use =
    coef.beta[,grep(coef.name, colnames(coef.beta))];}

  if(!is.null(dim(vcov.use)))
  {
    A.m = diag(dim(vcov.use)[1])
    beta.m = matrix(coef.beta.use, ncol = 1)
    val.chisqr.m  =  t(A.m%*%beta.m)%*%solve(t(A.m)%*%vcov.use%*%A.m)%*%(A.m%*%beta.m)
    pval = round(as.numeric(1-pchisq(val.chisqr.m, df=dim(vcov.use)[1])), digits=4)
```

© 2021 Gan T et al. *JAMA Network Open.*

```
    CI = matrix(NA, nrow = dim(vcov.use)[1], ncol = 3)
    for(lll in 1:dim(vcov.use)[1])
    {
      if (nominal){
        CI[lll, 1] <- exp((-1)*beta.m[lll,1])
        CI[lll, 2] <- exp((-1)*beta.m[lll,1] - abs(qnorm(0.025))*sqrt(vcov.use[lll,lll]))
        CI[lll, 3] <- exp((-1)*beta.m[lll,1] + abs(qnorm(0.025))*sqrt(vcov.use[lll,lll]))
      } else{
        CI[lll, 1] <- exp(beta.m[lll,1])
        CI[lll, 2] <- exp(beta.m[lll,1] - abs(qnorm(0.025))*sqrt(vcov.use[lll,lll]))
        CI[lll, 3] <- exp(beta.m[lll,1] + abs(qnorm(0.025))*sqrt(vcov.use[lll,lll]))
      }
    }
    CI = round(CI, digits=2)
    colnames(CI) <- c("point_estimate", "CI_lower", "CI_upper")
  }

  if(is.null(dim(vcov.use)))
  {
    val.chisqr.s = (coef.beta.use)^2/vcov.use
    pval = round(as.numeric(1-pchisq(val.chisqr.s, df=1)), digits=4)

    CI = matrix(NA, nrow = 1, ncol = 3)
    CI[1, 1] = exp(coef.beta.use)
    CI[1, 2] = exp(coef.beta.use - abs(qnorm(0.025))*sqrt(vcov.use))
    CI[1, 3] = exp(coef.beta.use + abs(qnorm(0.025))*sqrt(vcov.use))
    CI = round(CI, digits=2)
    colnames(CI) = c("point_estimate", "CI_lower", "CI_upper")
  }

  list.return = list()
  list.return[[1]] = pval
  list.return[[2]] = CI
  names(list.return) = c("p_value", "estimation")
  return(list.return)
}

check_PO <- function(vcov, coef.beta, coef.name)
{
  vcov.use = vcov[grep(coef.name, row.names(vcov)),grep(coef.name, colnames(vcov))]
  if(class(coef.beta)[1]=="numeric") {coef.beta.use =
    coef.beta[grep(coef.name, names(coef.beta))];}
  if(class(coef.beta)[1]=="matrix") {coef.beta.use =
    coef.beta[,grep(coef.name, colnames(coef.beta))];}

  if(!is.null(dim(vcov.use)))
  {
    dd = dim(vcov.use)[1]
    A.m = matrix(0, nrow=dd-1, ncol=dd)
    for (i in 1:(dd-1)){A.m[i,i] = 1; A.m[i,i+1] = -1}
    beta.m = matrix(coef.beta.use, ncol = 1)
    val.chisqr.m = t(A.m%*%beta.m)%*%solve(A.m%*%vcov.use%*%t(A.m))%*%(A.m%*%beta.m)
```

© 2021 Gan T et al. *JAMA Network Open.*

```
    pval = round(as.numeric(1-pchisq(val.chisqr.m, df=dd-1)),digits=4)
  }

  if(is.null(dim(vcov.use)))
  {
    pval = NA
  }
  return(pval)
}


library(ordinal)
nonPO.fit <- clm(TumorBudding ~ 1,
                 nominal = ~  BMI + Age + Gender + Race + Stage + TumorLocation  +
                 AppalachianStatus + TumorClusters + Desmoplasia + TumorBorder +
                 TumorStromaRatio + KMScore + Necrosis, data=data.use)
variables = c("BMI", "Age", "Gender", "Race", "Stage", "TumorLocation",
              "AppalachianStatus", "TumorClusters", "Desmoplasia", "TumorBorder",
              "TumorStromaRatio", "KMScore", "Necrosis")
PO.pval = matrix(nrow=length(variables),ncol=1)
rownames(PO.pval) = variables; colnames(PO.pval) = "p-value"
for (i in 1:length(variables)){
  PO.pval[i] = check_PO(vcov = vcov(nonPO.fit),
                        coef.beta = coef(nonPO.fit),
                        coef.name =  variables[i])
}

print(PO.pval)

partialPO.fit <- clm(TumorBudding ~ BMI + Age  + Race + TumorLocation  +
                 AppalachianStatus + TumorClusters + Desmoplasia + TumorBorder +
                 TumorStromaRatio,
                 nominal = ~  Gender + Stage + KMScore + Necrosis, data=data.use)
print(summary(partialPO.fit))

library(generalhoslem)
pulkrob.chisq(partialPO.fit, variables)
pulkrob.deviance(partialPO.fit, variables)

pvalue = NULL
for (i in 1:length(variables)){

  if (variables[i] %in% c("Gender", "Stage", "KMScore", "Necrosis")){
    pv.ci.fit <- Wald_pvalue_CI(vcov = vcov(partialPO.fit),
                                coef.beta = coef(partialPO.fit),
                                coef.name = variables[i], nominal = T)
  } else{
    pv.ci.fit <- Wald_pvalue_CI(vcov = vcov(partialPO.fit),
                                coef.beta = coef(partialPO.fit),
                                coef.name =  variables[i])
  }
print(variables[i])
print(pv.ci.fit)
```

© 2021 Gan T et al. *JAMA Network Open.*

```r
pvalue[i] = pv.ci.fit$p_value
}
names(pvalue) = variables
print(pvalue)

# Association between tumor budding and survival time (Figure 1 and Table 3)
library(survival)
library(survminer)
fit <- survfit(Surv(Survtime, Status) ~ TumorBudding, data=data.use1)
temp <- survdiff(Surv(Survtime, Status) ~ TumorBudding, data=data.use1)
thispvalue <- (1 - pchisq(temp$chisq, length(temp$n) - 1))
cat("Logrank P for comparing survival time across tumor budding groups:",
     round(thispvalue,digits=5), "\n\n")
setEPS()
postscript("figure2.eps")
ggsurvplot(fit, data=data.use1, risk.table = TRUE, pval=T,
              legend.labs=c("Low", "Intermediate", "High"),
              title="Kaplan-Meier Plot by Tumor Budding Grade",
              xlab="Time(years)", ylab="Alive (*100%)")
dev.off()

data12 <- data.use1[data.use1$TumorBudding==1 | data.use1$TumorBudding==2, ]
fit <- survfit(Surv(Survtime, Status) ~ TumorBudding, data=data12)
temp <- survdiff(Surv(Survtime, Status) ~ TumorBudding, data=data12)
thispvalue <- (1 - pchisq(temp$chisq, length(temp$n) - 1))
cat("Logrank P for comparing survival time between intermediate vs low tumor budding:",
     round(thispvalue,digits=4), "\n\n")

data13 <- data.use1[data.use1$TumorBudding==1 | data.use1$TumorBudding==3, ]
fit <- survfit(Surv(Survtime, Status) ~ TumorBudding, data=data13)
temp <- survdiff(Surv(Survtime, Status) ~ TumorBudding, data=data13)
thispvalue <- (1 - pchisq(temp$chisq, length(temp$n) - 1))
cat("Logrank P for comparing survival time between high vs low tumor budding:",
     round(thispvalue,digits=4), "\n\n")

data23 <- data.use1[data.use1$TumorBudding==2 | data.use1$TumorBudding==3, ]
fit <- survfit(Surv(Survtime, Status) ~ TumorBudding, data=data23)
temp <- survdiff(Surv(Survtime, Status) ~ TumorBudding, data=data23)
thispvalue <- (1 - pchisq(temp$chisq, length(temp$n) - 1))
cat("Logrank P for comparing survival time between high vs intermediate tumor budding:",
     round(thispvalue,digits=4), "\n\n")

fit.cox <- coxph(Surv(Survtime, Status) ~ TumorBudding + BMI + Age + Gender + Race + Stage +
                        TumorLocation  + AppalachianStatus, data=data.use)

cox.zph(fit.cox)

library(survMisc)
print(gof(fit.cox), maxCol=20)

variables = c("TumorBudding", "BMI", "Age", "Gender", "Race", "Stage", "TumorLocation",
              "AppalachianStatus")
for (i in 1:length(variables)){
```

© 2021 Gan T et al. *JAMA Network Open.*

```
  cox.pvalue.ci <- Wald_pvalue_CI(vcov = vcov(fit.cox),
                                  coef.beta  =  summary(fit.cox)$coefficients[  ,1],
                                  coef.name = variables[i])
  print(paste("For ", variables[i]))
  print(cox.pvalue.ci)
}
```

© 2021 Gan T et al. *JAMA Network Open.*
